# Malignancies masquerading as device pocket infections

**DOI:** 10.1016/j.hrcr.2021.07.009

**Published:** 2021-07-31

**Authors:** Toni Moseley, Ulrika Birgersdotter-Green, Gregory Feld, Travis Pollema

**Affiliations:** ∗ Division of Cardiothoracic Surgery, University of California San Diego, La Jolla, California; † Division of Cardiology, University of California San Diego, La Jolla, California

**Keywords:** Cancer, CIED, Device infection, Lead extraction, Malignancy, Pacemaker, Pacemaker complication, Pathology, Pocket infection

## Introduction

Infectious complications following cardiac implantable electronic device (CIED) implantation are associated with significant mortality. Establishing the correct diagnosis is important and not all presumed CIED pocket infections turn out to be infections. Rare cases of malignancy mimicking a pocket infection have been described. We present 2 cases of malignancy, initially thought to be a primary CIED pocket infection.

## Case report

### Case #1

**Figure 1:**
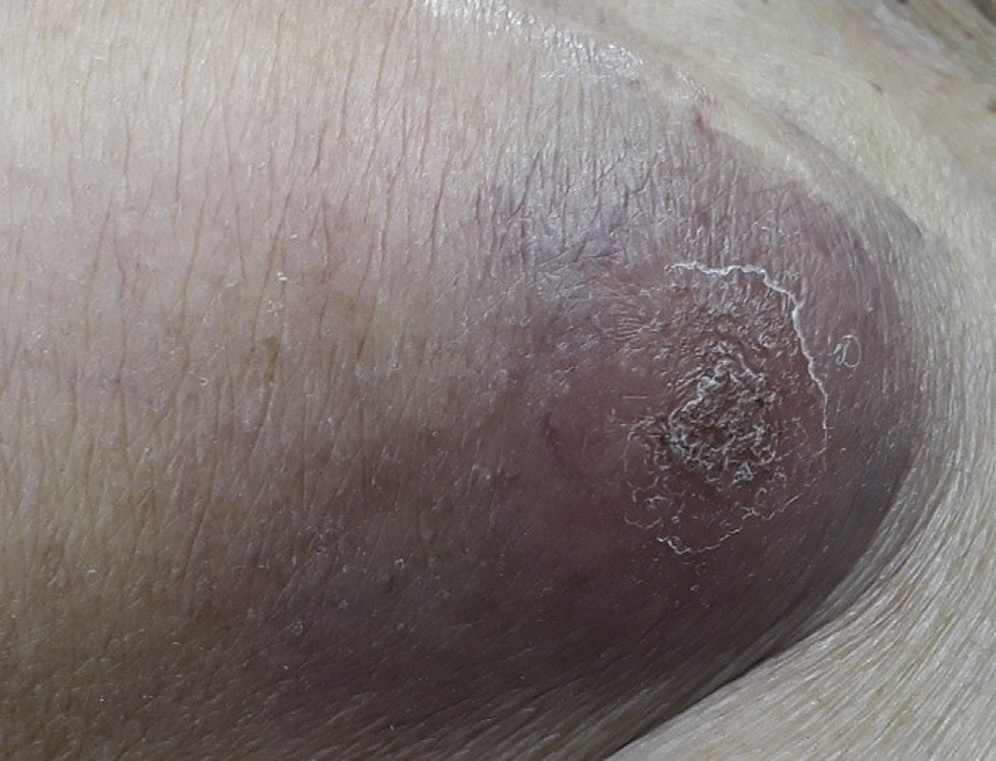
Case #1 at time of presentation.

**Figure 2:**
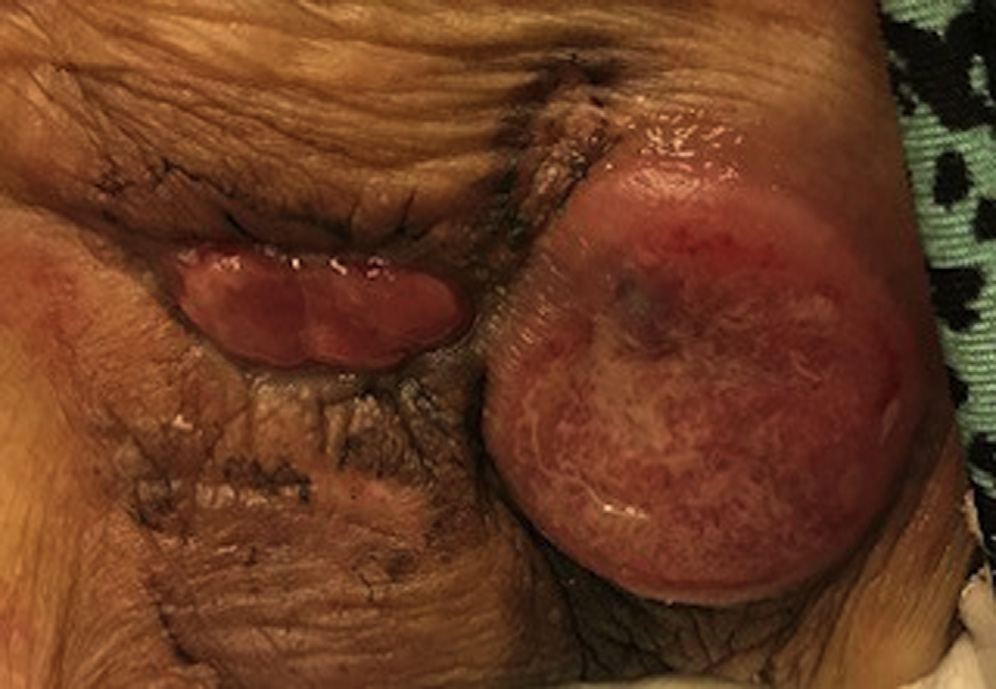
Case #1 wound 11 weeks after original surgery, just prior to return to operating room.

A 90-year-old woman was referred for management of CIED pocket infection. She had a history of complete heart block with pacemaker implantation in 2000 and cardiac resynchronization therapy defibrillator upgrade in 2014, end stage renal disease, hypertension, cerebrovascular accident, rheumatoid arthritis, diabetes mellitus, and remote lymphoma. She presented with 1 year of progressive left chest wall device pocket swelling, tenderness, and erythema. On examination the device site was swollen and tender to touch (Figure 1). She had no systemic signs or symptoms of infection. Her lab work did not reveal any hematologic abnormalities. An echocardiogram was performed with no evidence of endocarditis or lead vegetation. Management strategies were discussed with the patient and her family. Owing to her advanced age and multiple comorbidities, a lead extraction was not done and she underwent a CIED pulse generator removal, debridement, and complete capsulectomy. A new cardiac resynchronization therapy pacemaker device was placed in subpectoral fashion on the left side. At the time of the procedure an intense inflammatory reaction as well as necrosis was noted, but no purulence was seen. Swabs from the pocket as well as tissue from the capsule were sent for culture. The pocket was irrigated with hydrogen peroxide as well as antibiotic solutions and a negative-pressure wound therapy device was placed to assist with pocket closure. She recovered well and was discharged on a 3-week course of oral doxycycline. All cultures remained negative. An area of induration and discoloration persisted and increased in size, breaking through the epidermis and exposing vascularized tissue (Figure 2). Eleven weeks after her original surgery, she was brought back to the operating room for a pocket exploration, debridement, and primary wound closure. Tissue sent for pathology revealed large B-cell lymphoma. Given the patient’s remote history of B-cell lymphoma, this was felt to likely be a reoccurrence.

### Case #2

**Figure 3:**
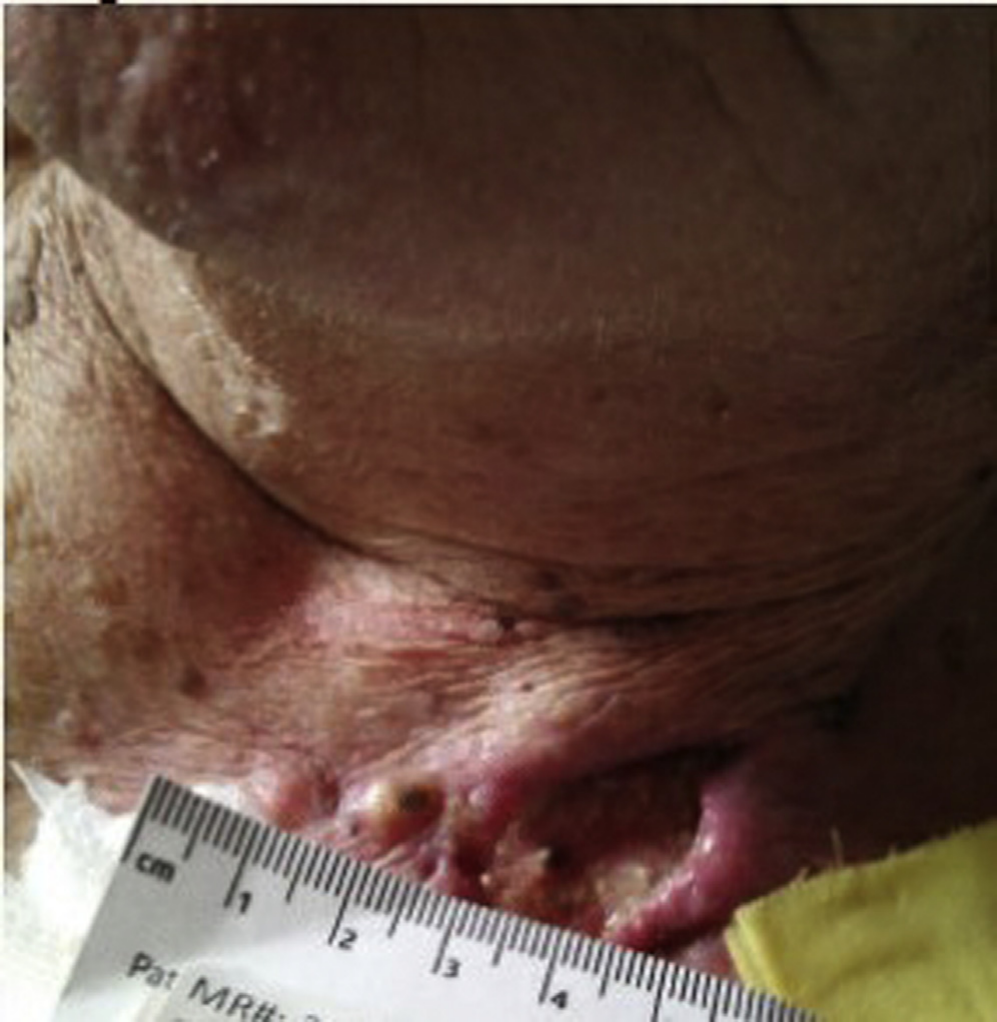
Case #2 at time of presentation.

An 84-year-old man was referred for management of a CIED pocket infection. He had a history of complete heart block with pacemaker in 1995, right ventricle lead revision in 2003, and a generator replacement in 2011. Past medical history was otherwise significant for permanent atrial fibrillation, diabetes mellitus, hypertension, and previous squamous cell carcinoma of his right forearm. He initially presented to an outside hospital with a “cyst” located in the left infraclavicular region. The “cyst” was excised and the wound left open to drain because of concern for infection. The wound culture was positive for *Staphylococcus aureus*. His wound was cared for by a dermatologist with topical ointments and systemic antibiotics for 4 months without wound healing. He was treated with vancomycin and piperacillin/tazobactam. He was referred for further management and lead extraction. Upon admission, he was afebrile and hemodynamically stable. His examination was significant for an open left upper chest wound measuring 5 × 2 cm, which was warm and tender to palpation and draining purulent fluid (Figure 3). The area was slightly superior to his pacemaker pocket, with concern for lead erosion. A left upper arm nodule was also noted. Wound cultures were positive for *Enterococcus faecalis* and *Pseudomonas aeruginosa*. Blood cultures were negative. An echocardiogram was negative for endocarditis or lead vegetation. Infectious disease was consulted, and they recommended continuation of piperacillin/tazobactam and device extraction because of its proximity to the infected wound and concern for involvement. Plastic surgery was consulted for wound evaluation prior to the device extraction. He underwent pulse generator removal and extraction of 3 permanent transvenous pacemaker leads without complication. His pocket contained a small amount of serous fluid, which was cultured, but otherwise there was no evidence of infection. A temporary-permanent pacemaker was placed via his right internal jugular vein. The plastic surgery team excised and debrided his clavicular wound and placed a split-thickness skin graft from his left thigh to the wound. After 72 hours of negative blood cultures, the patient underwent placement of a leadless pacemaker (Medtronic Micra™; Medtronic, Inc, Minneapolis, MN). He was discharged home to complete 2 weeks of intravenous antibiotic therapy with piperacillin/tazobactam. Pathology results from the wound debridement revealed moderately differentiated squamous cell carcinoma (SCC) extending into the deep margin and focally into the peripheral margins. He was referred to Oncology to discuss further management of the SCC. Prior to decision, a biopsy of the left upper arm nodule was performed to evaluate for disease in transit. The pathology results from an incisional biopsy were significant for B-cell lymphoma. He has undergone infusions to treat the lymphoma and continues to be followed by an oncologist for both his lymphoma and SCC.

## Discussion

The frequency of CIED implantation has increased significantly in the last 20 years, with the majority (70%) of implantations being done in patients >65 years of age and with the majority (75%) of those having 1 or more comorbidities.^1^ These patients may have remote medical problems, including a history of malignancy, that are not at the forefront of their current medical care. Along with this increase in CIED implantations, there is also a noted increase in device infections.^1^ CIED infections have been associated with a 2.4-fold increase in mortality when compared to patients without device infection, with female sex and impaired renal function predictors of a higher likelihood of developing device infection as well as increased mortality.^2^ Once a device infection has been recognized, system removal is recommended owing to unacceptably high rates of relapse and mortality when treated solely with antibiotics.^3^

It is, however, important to keep in mind that CIED pocket infections can sometimes be difficult to diagnose and distinguish from other pathologic processes. Pain and tenderness at the device site may represent a wide range of clinical scenarios from an underlying infection to possible CIED allergies or musculoskeletal problems. It seems to be well understood that subclinical infection is a common cause of the early presentation of pain following device placement, but it has also been linked to cases of chronic pain after device placement.^4^^,^^5^ In addition, the clinical presentation of a device infection is often variable, ranging from subclinical device infections to a straightforward presentation with localized pocket swelling associated with fever and leukocytosis. A case series by Korantzopoulos and colleagues^6^ reported 5 instances of skin lesions over cardiac device pockets that mimicked infection. The reported cases were found to be caused by localized cellulitis, herpes zoster, spontaneous hematoma, and irritant contact dermatitis.

There is an extremely low incidence of malignancy associated with cardiac devices, with less than 25 reported in the literature. A case report and literature review by Zarifi and colleagues^7^ reported a single case of large B-cell lymphoma arising from a CIED pocket along with a review of 15 previous reported cases of malignancy arising from CIED sites from 1976 to 2013. These reported cases were due to a wide variety of malignancies, including various types of breast cancer, adenocarcinoma, plasmacytoma, and sarcomas. De Mattia and colleagues^8^ presented images showing a lesion growing below a pacemaker pocket at the superior portion of a patient’s breast that was concerning for granuloma. Ultrasound and mammography images led to a suspicion for breast cancer, which was proven by biopsy. An additional 3 case reports of large B-cell lymphoma associated with cardiac devices all note presenting symptoms that were suggestive of an infectious process, including a case of tumor mimicking lead vegetation on echocardiogram.^9^, ^10^, ^11^ Snorek and colleagues^12^ presented a case report in which a patient with a history of chronic lymphocytic leukemia presented with painless erythema and pruritus in a pocket area as well as mildly elevated inflammatory blood markers (C-reactive protein 17.3 ng/L and procalcitonin 0.2 ng/mL). Because of the atypical presentation, device extraction was not done, and instead an exploratory incision was done with culture and pathology samples were sent. Histological findings were consistent with low-grade B-cell chronic lymphocytic leukemia/small lymphocytic lymphoma.^12^

Multiple theories have been proposed regarding the potential oncogenicity of pacemakers, including chronic inflammation from device pockets leading to development of lymphoma cells.^7^^,^^11^ Of all case reports of malignancy associated with CIEDs, there are only 4 previously noted cases of lymphoma. Our first case seems to be the fifth lymphoma to be reported. In case #2 the simultaneous finding of a large B-cell lymphoma in a left arm nodule is intriguing but of unclear significance. A single case of SCC and large B-cell lymphoma occurring together has been reported, with both occurring in the head and neck region.^13^

Sensitivity to the various types of metals used in CIEDs have also been noted in the literature to present in patients similar to that of infection with pain, erythema, and warmth.^14^ In a review of allergies to surgical implants, Pacheco discussed the need for preimplantation patch testing in patients with known allergies to various metal components. In a patient without a previous history of metal allergy, consideration of postimplant patch testing is suggested to only be done once more common causes of symptoms (such as infection) have been ruled out.^14^

In our first patient, while there were no signs of a systemic infection, the findings associated with the CIED pocket were felt to be suspicious for an underlying infection. In retrospect, the operative observation of “intense inflammatory reaction,” as well as necrosis but no purulence, should have prompted tissue to be sent to pathology. In our second patient, there was clear, documented infection in tissue adjacent to the device, leading to an appropriate device and lead removal. The overall clinical scenario did, in this case, lead to a tissue pathology examination revealing the underlying cause as squamous cell carcinoma.

## Conclusion

Not all presumed CIED pocket infections turn out to be true primary pocket infections. Rare cases of malignancy mimicking a CIED pocket infection have been described. Both cases presented here had a malignancy in their remote past medical histories and likely represented reoccurrence of previous disease, not primary disease, as found in previous case reports. These 2 cases demonstrate the importance of awareness of comorbidities and atypical clinical and/or operative presentation, which should prompt an attempt at an early tissue diagnosis and collaboration across specialties. It is important to consider that what may appear as an infection could be other processes mimicking infection.

**Key Teaching Points**

• Not all presumed cardiac implantable electronic device (CIED) pocket infections are true primary infections.
• In patients with a history of hematological disease or malignancy, it is important to identify and exclude cutaneous manifestations of the disease prior to device system removal.
• Tissue should be sent to pathology when uncertain of etiology.
• A multidisciplinary treatment team including surgery, cardiac electrophysiology, infectious disease, plastic surgery, and oncology is important when encountering unusual cutaneous findings associated with CIEDs.
